# hMSCs treatment attenuates murine herpesvirus-68 (MHV-68) pneumonia through altering innate immune response via ROS/NLRP3 signaling pathway

**DOI:** 10.1186/s43556-023-00137-z

**Published:** 2023-09-14

**Authors:** Aiping Qin, Xiao-juan Wang, Jijun Fu, Ao Shen, Xiaotao Huang, Zhida Chen, Huiting Wu, Yu Jiang, Qian Wang, Fei Chen, Andy Peng Xiang, Xiyong Yu

**Affiliations:** 1 Guangzhou Municipal and Guangdong Provincial Key Laboratory of Molecular Target & Clinical Pharmacology, the NMPA and State Key Laboratory of Respiratory Disease, School of Pharmaceutical Sciences and the Fifth Affiliated Hospital, Guangzhou Medical University, Guangzhou, 511436 China; 2 Institute of Urology, The Third Affiliated Hospital of Shenzhen University (Luohu Hospital Group), Shenzhen, 518000 China; 3 Guangzhou Municipal and Guangdong Provincial Key Laboratory of Protein Modification and Degradation, School of Basic Medical Science, Guangzhou Medical University, Guangzhou, 511436 China; 4 Center for Stem Cell Biology and Tissue Engineering, Key Laboratory for Stem Cells and Tissue Engineering, Ministry of Education, Sun Yat-sen University, Guangzhou, 510080 Guangdong China

**Keywords:** Mesenchymal stem cell, Murine gammaherpesviruses 68, Pneumonia, Innate immune, NLRP3 inflammasome

## Abstract

Immunocompromised individuals are particularly vulnerable to viral infections and reactivation, especially endogenous herpes viruses such as Epstein-Barr virus (EBV), a member of oncogenic gamma-herpesviruses, which are commonly linked to pneumonia and consequently significant morbidity and mortality. In the study of human and animal oncogenic gammaherpesviruses, the murine gamma-herpesviruses-68 (MHV-68) model has been applied, as it can induce pneumonia in immunocompromised mice. Mesenchymal stem cell (MSC) treatment has demonstrated therapeutic potential for pneumonia, as well as other forms of acute lung injury, in preclinical models. In this study, we aim to investigate the therapeutic efficacy and underlying mechanisms of human bone marrow-derived MSC (hMSC) on MHV-68-induced pneumonia. We found that intravenous administration of hMSCs significantly reduced lung damages, diminished inflammatory mediators and somehow inhibited MHV-68 replication. Furthermore, hMSCs treatment can regulate innate immune response and induce macrophage polarization from M1 to M2 phenotype, could significantly alter leukocyte infiltration and reduce pulmonary fibrosis. Our findings with co-culture system indicated that hMSCs effectively reduced the secretion of of inflammation-related factors and induced a shift in macrophage polarization, consistent with in vivo results. Further investigations revealed that hMSCs treatment suppressed the activation of macrophage ROS/NLRP3 signaling pathway in vivo and in vitro. Moreover, administration of MCC950, a selective NLRP3 inhibitor has been shown to effectively reduce ROS production and subsequently alleviate inflammation induced by MHV-68. Taken together, our work has shown that hMSCs can effectively protect mice from lethal MHV-68 pneumonia, which may throw new light on strategy for combating human EBV-associated pneumonia.

**Supplementary Information:**

The online version contains supplementary material available at 10.1186/s43556-023-00137-z.

## Introduction

Epstein–Barr virus (EBV) infection is prevalent among healthy individuals, and most cases are either asymptomatic or self-limiting in nature. However, in immunocompromised individuals, such as HIV patients, solid organ and bone marrow allograft recipients or patients receiving immune-suppressive drugs, EBV is capable of establishing latent-recurrent infections, and giving rise to disease in both the acute and recurrent phases. This is associated with several important lymphomas, specifically Burkitt’s, Hodgkin’s disease and immunoblastic lymphoma [1], fever and end-organ diseases, e.g. encephalitis/myelitis, hepatitis, pneumonitis and myocarditis [2–4]. Notably, the lung is a major site of EBV infection in immunocompromised patients [4, 5]. Currently, preemptive therapy for these patients is primarily relied on reducing immunosuppressants, adoptive cellular therapy (EBV-specific CTL) and B-cell depletion with monoclonal antibodies (e.g. rituximab) or combined chemotherapy. Still, the effectiveness of these treatments is not ideal [6]. For the purpose of developing treatments for EBV and other gammaherpesviruses, the murine gamma-herpesviruses-68 (MHV-68) model has been applied as an ideal model, since it could cause pneumonitis in immunocompromised mice and efficiently completes the lytic phase and productively infects cultured cells [7].

As an adoptive cellular therapy, mesenchymal stem cells (MSCs) have shown promising therapeutic potential for tissue damage, and various inflammatory and autoimmune diseases, owing to their capacity for self-renewal, multipotency, and potent immunomodulatory properties [8, 9]. Our group and others have demonstrated that human or mouse MSCs can improve survival from sepsis mice, alleviate inflammation of intestinal and experimental colitis, enhance homing, and especially exert therapeutic effects in acute graft versus host disease (aGVHD) and chronic GVHD (cGVHD) patients [9–16]. Recent studies have also shown that MSCs can protect infected tissues from brain, liver and lung injury caused by various viruses, including Japanese encephalitis virus, hepatitis C virus, and respiratory viruses such as H1N1 (SwIV), H5N1, H9N2 AIV and SARS-CoV-2 [16–19]. The therapeutic effect of MSCs in acute lung injury has been shown to facilitate tissue regeneration, homing to injury sites, decreasing respiratory inflammation, restoring alveolar fluid balance and enhancing the function of immune cells [8–11, 16–19]. Pre-clinical and clinical data also support that the treatment of human MSCs (hMSCs) could decrease the EBV titers from serum or lung bronchoalveolar lavage (BAL) in transplant patients, though statistically, it makes little difference [20, 21]. It will, however, hold great promise in understanding the role of hMSCs in EBV infection, especially EBV- induced pneumonia. In addition, the molecular mechanisms through which hMSCs augment anti-EBV infection remain unclear.

Upon EBV infection, innate immune response serves as the first line of defense against pathogens. Dendritic cells (DCs), monocytes/macrophages, neutrophils, and natural killer (NK) cells are specialized in sensing pathogens and producing cytokines. These cells play a crucial role not only in direct antiviral properties but also for the proper formation of adaptive immune response [22, 23]. During the early stage of EBV infection, innate immune response is substantially mounted, accompanied by the activation of inflammatory signalling pathways in the host cell. As a cell in almost all tissues, macrophages play important roles in maintaining tissue homeostasis and have a central role in inflammation and host defense [23]. NK cells are also crucial in early control of EBV infection, as they are involved in controlling intracellular pathogens and tumours. They are able to kill transformed and infected cells directly, and also produce cytokines that stimulate other components of immune system [24, 25]. For MHV-68, immediate-early gene RTA (encoded by ORF50) is an important transcriptional activator and sufficient to initiate lytic replication from latently-infected cells [26, 27]. Importantly, as a promising stem cell, MSCs play an important role in regulating the function of innate and acquired immune cells [8–16]. However, whether MSC handles the innate immune system infected by EBV or not, and through what mechanism requires further studies.

In this study, we established an MHV-68 –induced pneumonia model in BALB/c-nu mice to study the in vivo host interaction of human EBV and to develop therapeutic or preventive strategies against EBV- associated pneumonia.

## Results

### Histological evaluation of MHV-68 pneumonia and viral replication

We first established a mouse model for MHV-68 interstitial pneumonia, and evaluated the pulmonary MHV-68 replication from infected BALB/c-nu mice. As shown in Supplementary Fig. 1a-d, infection of MHV-68 resulted in a significantly increased expression of MHV-68-luciferase protein (Supplementary Fig. 1a). The expression of immediate-early gene ORF50 (Supplementary Fig. 1b), as well as mature virus particles as measured by PFU (Supplementary Fig. 1d) were all increased on day 7 and day 14 post-infection. Histological analysis showed lung tissues from MHV-68-infected mice were markedly impaired compared with 0 day mice and displayed characteristics of interstitial pneumonia, i.e. pulmonary interstitial edema, the alveolar walls had become thickened and caused focal, diffuse diminution or obliteration of alveolar space (Supplementary Fig. 1c).

### hMSCs treatment improved survival and reduced lung injury in MHV-68 pneumonia

Human MSC (hMSC) cultures were isolated and cultured from bone marrow aspirates of healthy voluntary donors as previously described [28]. Before transplantation, hMSCs were assessed for surface marker profile and differentiation potential into adipocytes and osteocytes (Supplementary Fig. 2a-c).

**Fig. 1:**
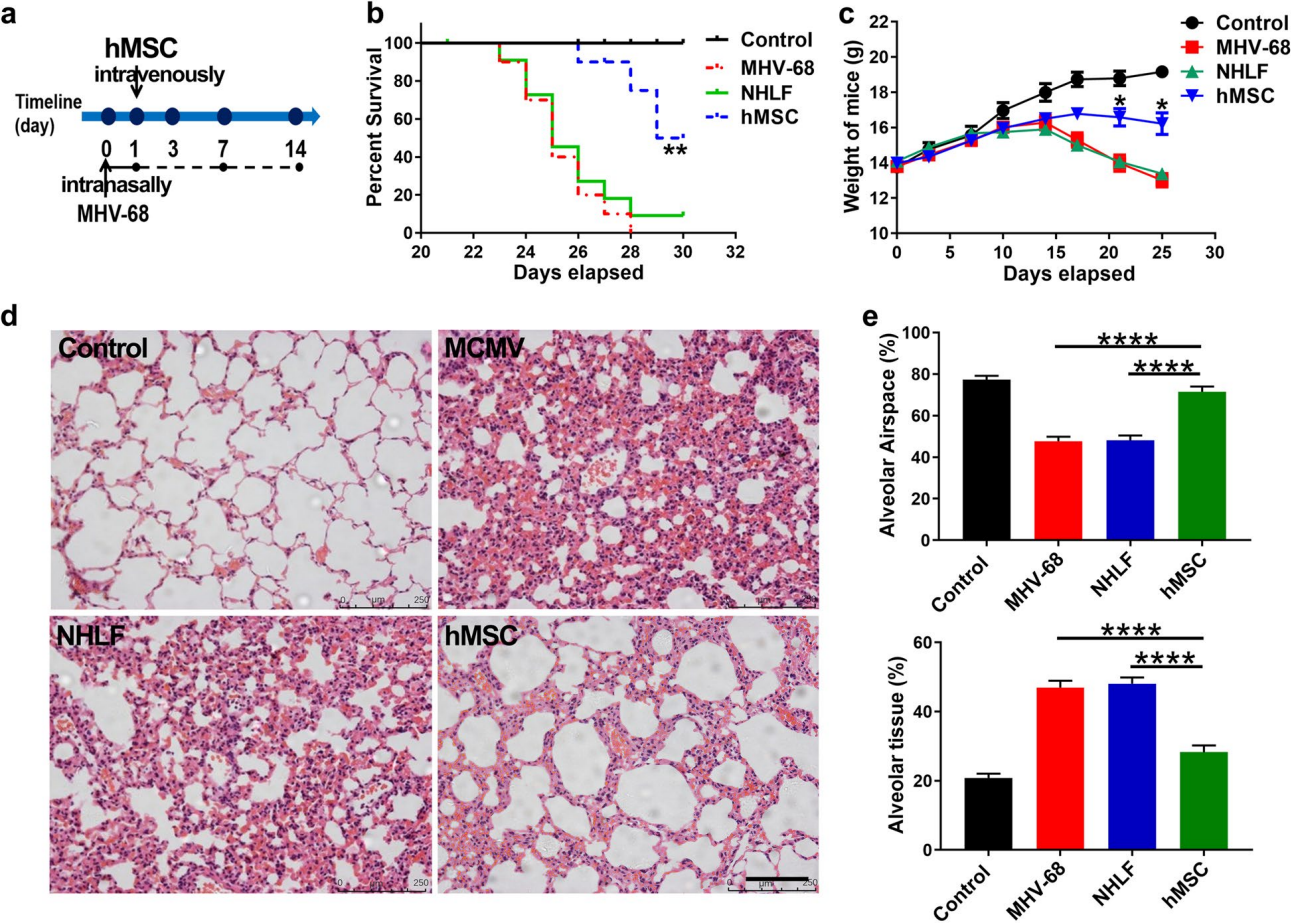
hMSCs treatment alleviates MHV-68-induced pneumonia in BALB/C-nu mice. Mice were infected intranasally with 2 × 10^5^ PFU of MHV-68. **a** Experimental strategy of hMSCs infusion. **b** Survival curve of hMSCs treated mice versus other treatment groups, up to 30 day after Intranasal instillation of MHV-68 (**P < 0.01 vs. NHLFs group, *n* = 10). **c** Weight curves of mice in different experimental groups are shown (*P < 0.05 vs. NHLFs group, *n* = 10). **d** Lung pathology of mice in different experimental groups at 14 days post-infection (hematoxylin and eosin, 100× magnification). **e** Alveolar tissue percentage and alveolar airspace percentage in “e” picture were shown by analyzing lung sections from different experimental groups, *n* = 6. Control: Control group; MHV-68: MHV-68 + PBS group; NHLFs: MHV-68 + NHLFs group; hMSCs: MHV-68 + hMSCs group. Data are expressed as the means ± SEM. *P < 0.05; **P < 0.01; ****P <0.0001; N.S., not significant

To determine whether exogenous hMSCs protect against the development of MHV-68-induced pneumonia, survival rate, body weights and histological scores from infected mice were analyzed at series time points as indicated. As shown in Fig. 1a and b, MHV-68-induced pneumonia kill all infected mice within 30 days compared with the 100% survival rate (10 of 10) of the uninfected control (PBS) group. While transplantation of normal human lung fibroblasts (NHLFs) show no significance effect on the survival of MHV-68-infected mice, transplantation of hMSCs significantly improved the survival rate of MHV-68-infected mice (70%, 7 of 10 mice surviving, *p* < 0.01 vs NHLFs group, *p* < 0.001 vs none-transplantation MHV-68 group) (Fig. 1b). Body weights of infected mice after hMSCs treatment were marginally higher than in the MHV-68 and NHLF group and were significantly greater on day 20 and 25 post-infection (day 20, *p* < 0.05; day 25, *p* < 0.05, Fig. 1c). Histologic analysis showed that the increased alveolar congestion, haemorrhage, inflammatory cell infiltration, and wall thickening observed 14 day after MHV-68 inoculation was significantly attenuated with intravenous transplantation of hMSCs, but not with NHLFs transplantation (Fig. 1d and e).

### hMSCs transplantation reduced pulmonary inflammation in MHV-68 pneumonia mice

**Fig. 2:**
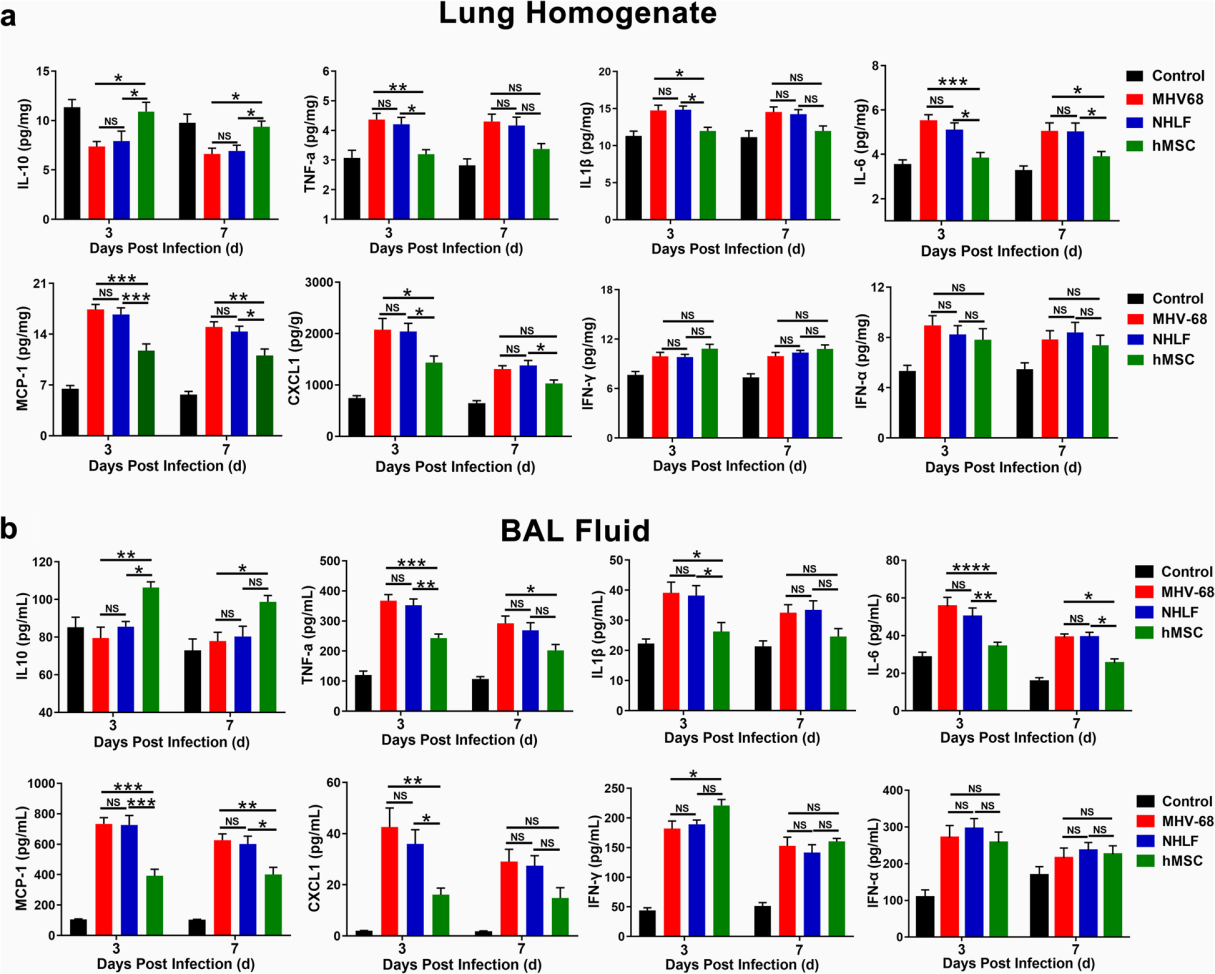
Effects of hMSCs on pulmonary cytokine and chemokine production. The levels of IL-10, TNFα, IL-1β, IFN-γ, IFN-α, MCP-1 and CXCL1 in lung homogenate (**a**), and BAL fluid (**b**) were detected by ELISA. Data are expressed as mean ± SEM, *n* = 6. *P < 0.05; **P < 0.01; ***P < 0.001; ****P <0.0001; N.S., not significant

Intranasal instillation of MHV-68 resulted in a significant inflammation response in the infected alveoli (Fig. 2). At 3 days post-infection, intravenous administration of hMSCs significantly decreased the concentrations of pro-inflammatory cytokines and chemokines, i.e. TNF-α, IL-1β, MCP-1, CXCL1 in lung homogenate and/or BAL fluid, respectively, as compared with mice treated with NHLFs group (Fig. 2a and b). In the meantime, anti-inflammatory cytokine IL-10 was significantly increased at 3 and 7 days post-infection. Interestingly, the level of IFN-γ, which is a critical cytokine for resistance against acute viral infection [29], in lung homogenate and BAL fluid were slight upward trend, compared with the NHLFs-treated group (Fig. 2a and b). Whereas the level of IFN-α, which is also important for antiviral responses, was no significant difference between hMSCs group and NHLFs group. These results suggest that hMSCs administration can ameliorate lung inflammation from MHV-68 infected mice.

### Antiviral effect of hMSCs transplantation

**Fig. 3:**
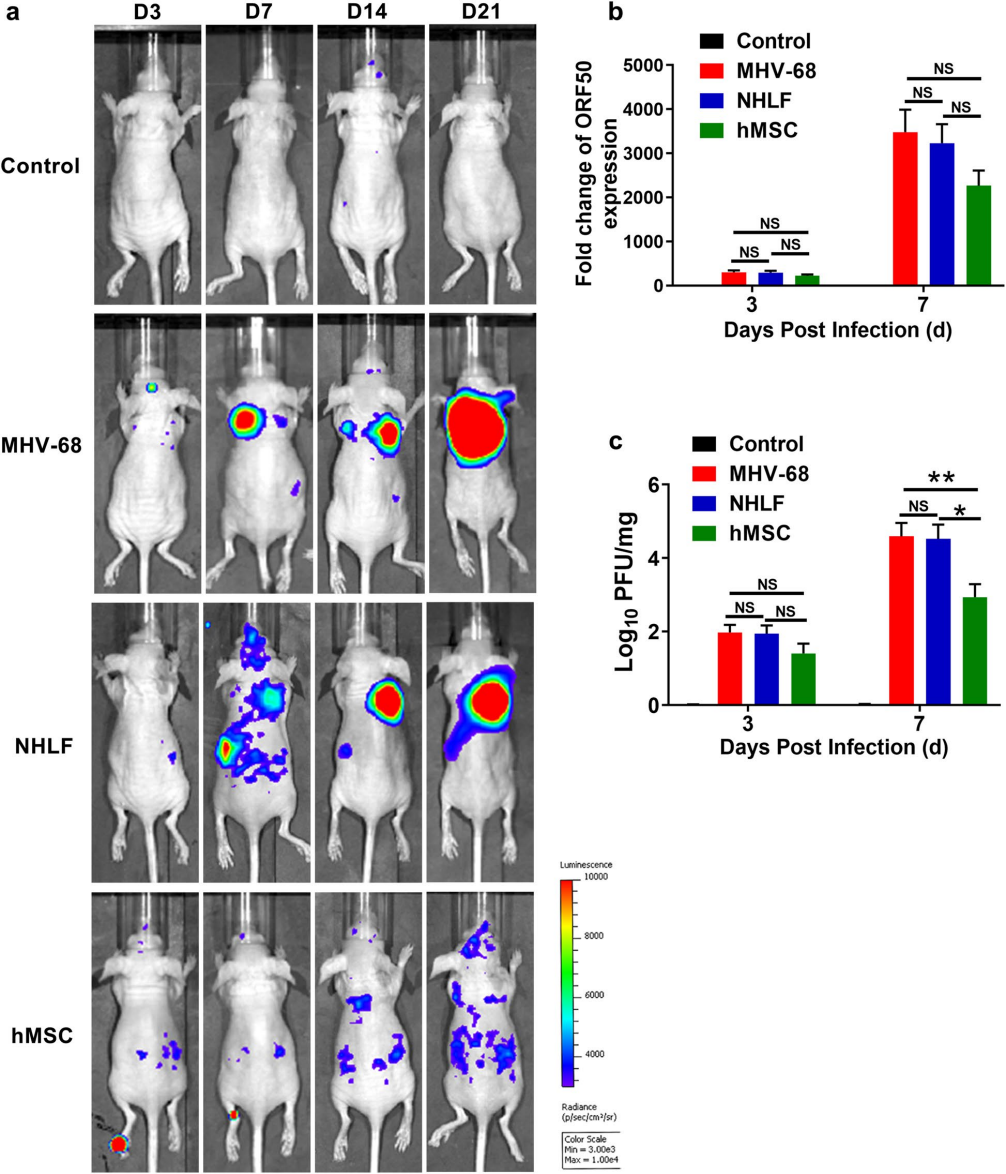
Antiviral effect of hMSCs treatment. Mice were infected intranasally with 2 × 10^5^ PFU of MHV-68. **a** The luciferase M3FL images at different time points are shown to represent the progression of MHV-68 replication. **b** The expression of pulmonary ORF50 gene from control and MHV-68 infected-group were measured by RT-PCR. **c** MHV-68 viral load from BAL fluid were measured by PFU assay at day 3 and day 7 post-infection. Data are expressed as mean ± SEM, *n* = 6. *P < 0.05; **P < 0.01; N.S., not significant

Since hMSCs administration reduces lung injury of MHV-68-infected mice, we next evaluated it effects on MHV-68 replication. Using an IVIS imaging system, we monitored the MHV-68 replication via the luciferase from the firefly luciferase expression cassette (M3-FL). At the indicated times post-infection, the M3-FL luminescence signals were significantly reduced in hMSCs-treated group, but not NHLFs-treated group, compared with the MHV-68-infected group (Fig. 3a). Then the expression of viral gene ORF50 and mature virus particles from BAL fluid were measured by RT-PCR and PFU assay at day 3 and day 7 post-infection, respectively (Fig. 3b and c). A downward trend in virus loads in hMSCs-treat group was observed, particularly by PFU assay, compared with the MHV-68 infected control and NHLF-treated group. These results revealed that hMSCs administration decreased the MHV-68 replication in immunocompromised mice.

### hMSCs transplantation modulated the innate immune response

**Fig. 4:**
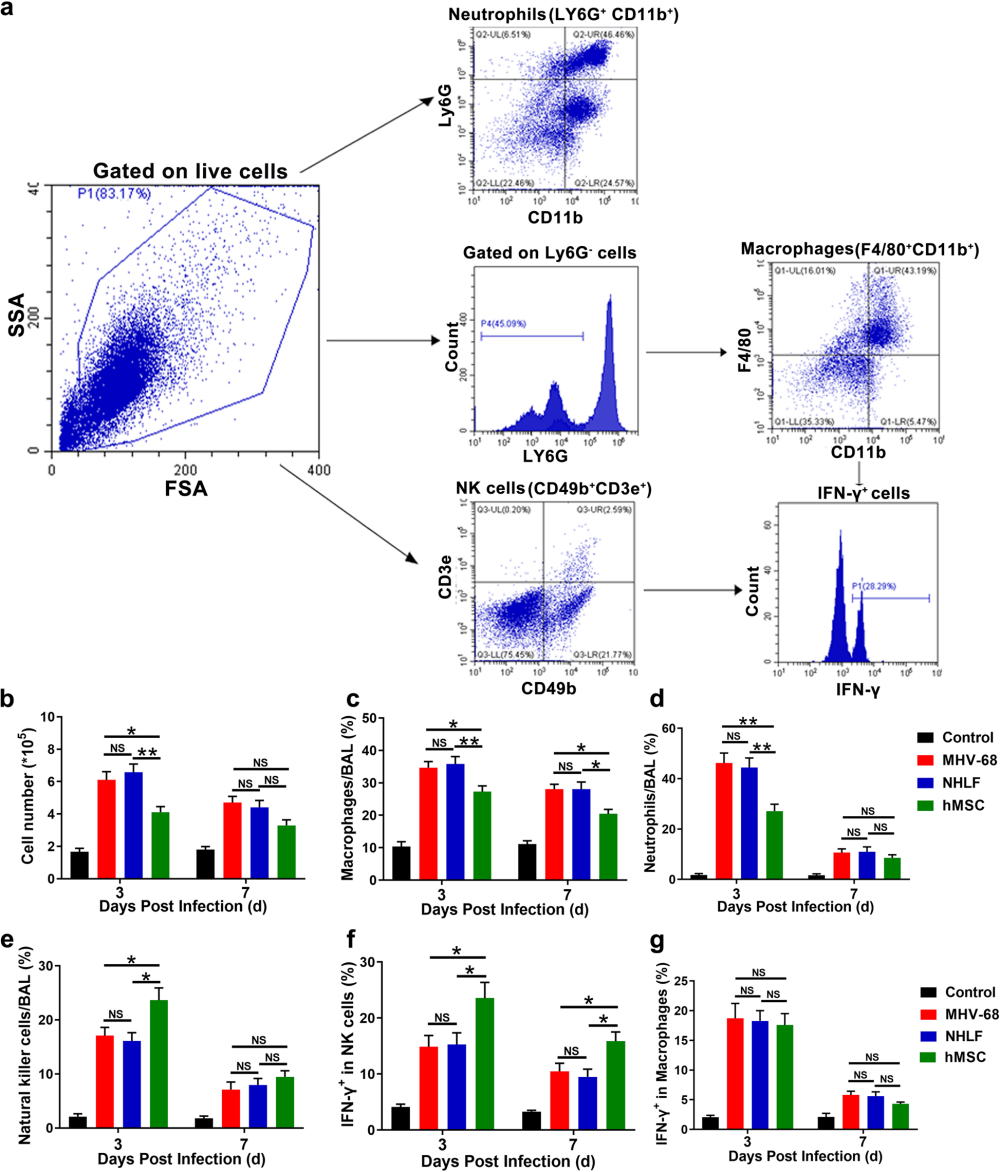
Immunomodulatory effect of hMSCs on innate immune cells. Mice were infected intranasally with 2 × 10^5^ PFU of MHV-68. Leukocytes from BAL fluid were analyzed by flow cytometry. **a** Gating strategy of Ly6G^-^CD11b^+^F4/80^+^ macrophage, Ly6G^+^CD11b^+^ neutrophil and CD3e^-^ CD49b^+^ natural killer (NK) cells. The total number of leukocytes (**b**), macrophages (**c**), neutrophils (**d**) and NK cells (**e**) were measured. The expression of intracellular IFN-γ in NK cells (**f**) and macrophages (**g**) were measured and analyzed by intracellular staining method. Data are expressed as mean ± SEM, *n* = 6. *P < 0.05; **P < 0.01; N.S., not significant

To investigate the effects that hMSCs administration acts on the MHV-68 infected mice, we look on innate immunity. Intranasal instillation of approximately 2 × 10^5^ MHV-68 resulted in severe lung injury, characterized by an influx of white blood cells in the infected alveolus (Fig. 4a and b). Intravenous administration of hMSCs reduced the influx of white blood cells by 28% (Fig. 4b), macrophages by 22% (Fig. 4c) and neutrophils by 39% (Fig. 4d) in the BAL fluid on day 3 post-infection compared with NHLFs-treated mice. The decrease of macrophages lasted until day 7 post-infection. In contrast, natural killer (NK) cells in the BAL fluid showed a statistically significant increase on day 3 post-infection after hMSCs transplantation (Fig. 4e). No therapeutic effect was observed with the intravenous administration of the same quantity of NHLFs.

Since we have shown that IFN-γ tended to increase after hMSCs transplantation in MHV-68-infected mice (Fig. 2a and b), we try to locate its origin. Flow cytometry data revealed that IFN-γ producing NK cells increased significantly after hMSCs infusion, compared to NHLF-infusion group, while the production of IFN-γ in macrophages displayed almost no changes (Fig. 4f and g). These results suggested that the antiviral effect of hMSC at least be partly through increasing the production of IFN-γ by NK cells.

### hMSCs transplantation modulate macrophage polarization in vitro and in vivo

**Fig. 5:**
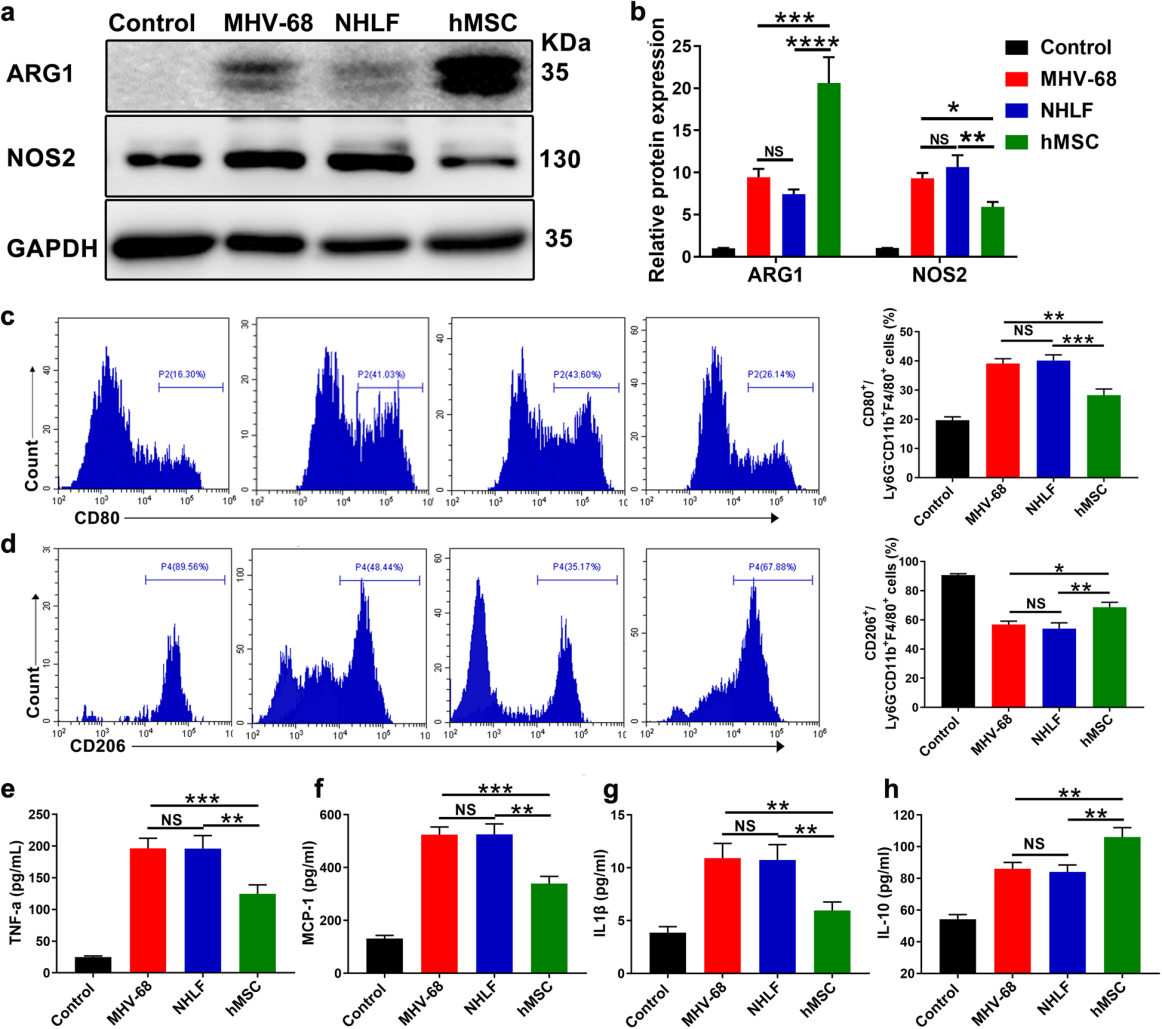
hMSCs regulated macrophage polarization in MHV-68-infected mice. Mice were infected intranasally with 2 × 10^5^ PFU of MHV-68. **a** & **b** Western blot was used to analyze the expression of NOS2 and ARG1 in lung homogenate at day 3 post-infection. The expression of CD80 (**c**) and CD206 (**d**) gated on Ly6G^-^CD11b^+^F4/80^+^ cells in BAL fluid were assayed by flow cytometry (Left, representative flow cytometry data; Right, statistical analysis results). Data are expressed as mean ± SEM, *n* = 6. **e** - **h** The expression of TNF-α (**e**), MCP-1 (**f**), IL-1β (**g**) and IL-10 (**h**) in supernatant from co-cultured BMDMs were measured by ELISA. Data are expressed as mean ± SEM, *n* = 4. *P < 0.05; **P < 0.01; ***P < 0.001; ****P < 0.0001; N.S., not significant

Macrophages and monocytes play an important role in producting inflammatory mediators during bacterial or viral infection and seem to be a major cell target in MHV-68 infection [1, 2, 30]. Many reports have shown that MSCs relieve inflammation and adjust cytokines by regulating macrophage polarization in various inflammatory models [8, 9]. We next assessed the potential of hMSCs to regulate macrophage polarization in vivo and in vitro. Firstly, we detected the expression of ARG1 and NOS2, the well-known macrophage phenotype M2 and M1 markers, in lung homogenate on day 3 post-infection by western blotting. A significantly increase in ARG1 expression was observed after hMSCs infusion, accompanied with a significant decrease in NOS2 expression (Fig. 5a and b). Flow cytometry data also assess expression of pulmonary M1/M2 markers (significantly decreased M1 CD80 and significant increased M2 CD206) after MHV-68 infection (Fig. 5c and d).

Further, in vitro experiments were carried out using transwell system. hMSCs or NHLFs were co-cultured for 48h with bone marrow-derived macrophages (BMDMs) after MHV-68 infection (MOI = 0.05). The results showed consistent with the in vivo results, that was increased CD206 and ARG1 expression and decreased CD80 and NOS2 expression (Supplementary Fig. 3a-d). In addition, we also detected the expression of cytokines and chemokines from the supernatant of MHV-68-infected BMDMs in the co-culture transwell system. The level of TNF-α, IL-1β and MCP-1 was decreased and the level of IL-10 was increased in hMSCs co-culture groups, suggesting an anti-inflammatory effect, consistent with the effects of MSC infusion in vivo (Fig. 5e, f, g and h). These results revealed MSC treatment could alleviate lung inflammatory via regulating macrophage polarization.

### hMSCs transplantation reduces pulmonary fibrosis in MHV-68-infected mice

**Fig. 6:**
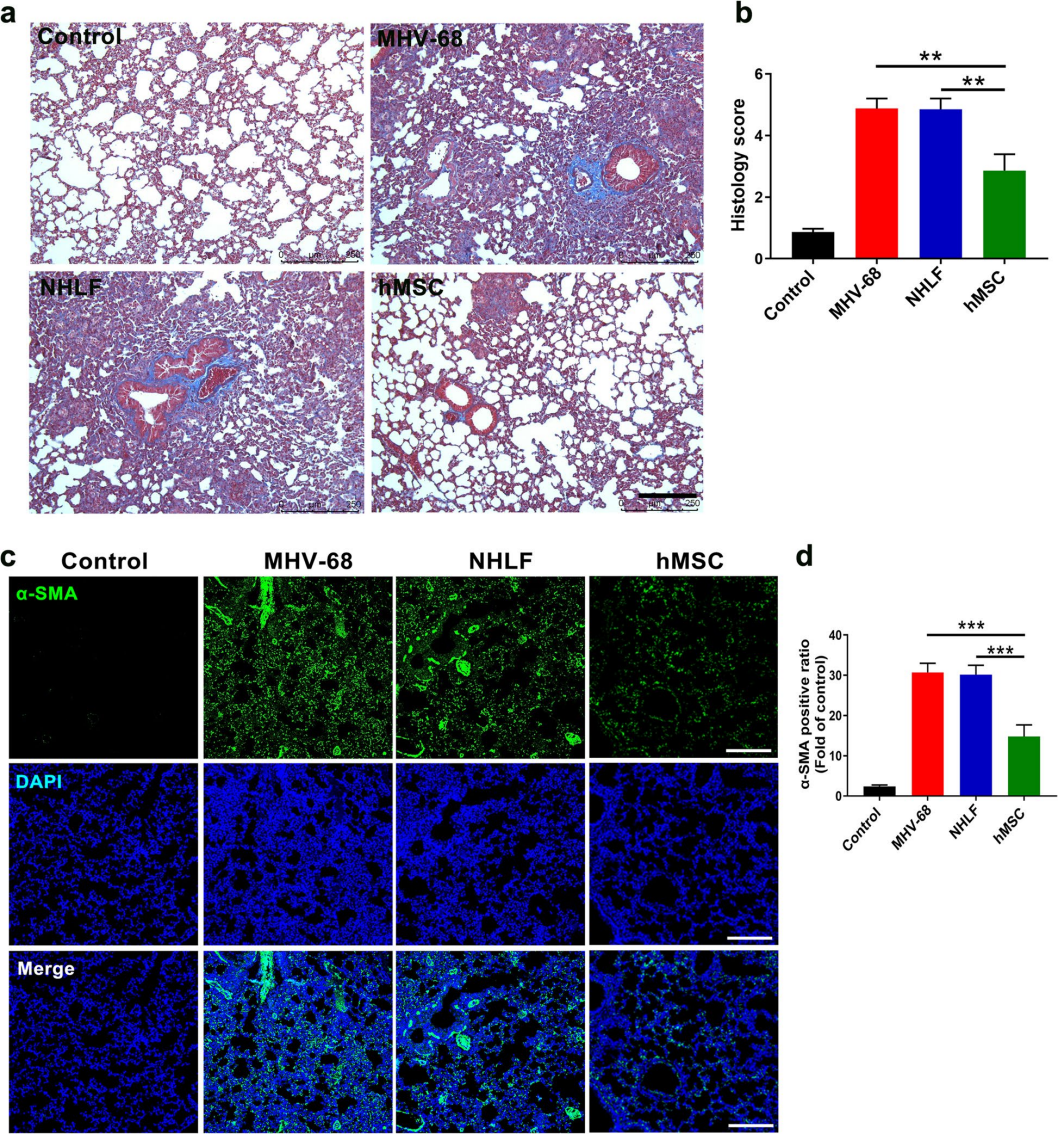
hMSCs reduces pulmonary fibrosis in MHV-68-infected mice. **a** Representative lung staining with Masson’s trichrome from MHV-68-infected mice at 14 days post-infection after hMSCs or NHLFs infusion. **b** Quantitative analysis of Masson staining by image J software. The blue staining represents deposition of collagen. Scale bars, 200 μm. **c** The protein levels of α-smooth muscle actin (α-SMA) (green) in lung tissues was measured by immunofluorescence staining at 14 days post-infection. **d** Quantification of α-SMA positive cells. DAPI was used to nuclear staining (blue). Scale bars, 200 μm. Data are expressed as mean ± SEM, *n* = 6. **P < 0.01; ***P < 0.001; N.S., not significant

Various viral infections, including those caused by herpesviruses, are associated with pathological fibrosis processes [31]. We thus evaluated the degree of pulmonary fibrosis in MHV-68-infected mice. We found hMSCs administration significantly attenuated the severity of pulmonary fibrosis confirmed by morphological changes assessed by Masson’s trichrome staining on day 14 post-infection (Fig. 6a and b). Moreover, the protein level related to fibrosis, α-smooth muscle actin (α-SMA) was profoundly decreased in hMSCs-infusion group, compared to NHLFs-infusion group by immunofluorescence staining (Fig. 6c and d).

### hMSCs transplantation inhibits the activation of NLRP3 signalling in vivo and in vitro

**Fig. 7:**
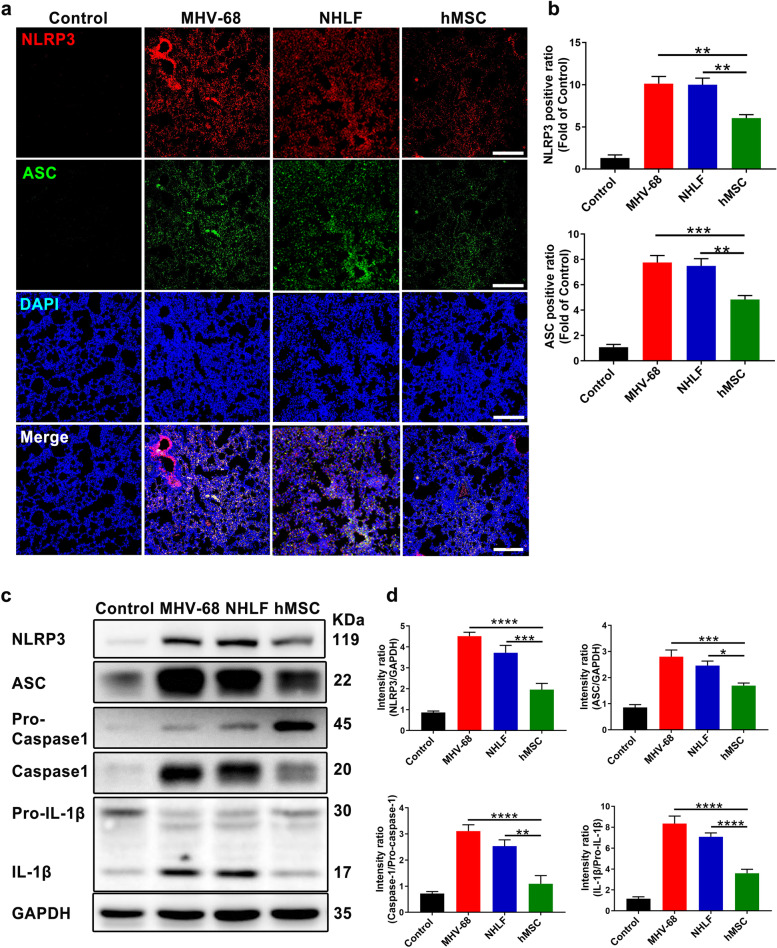
hMSCs reduces the activation of NLRP3 signaling in MHV-68-infected mice. Mice were infected intranasally with 2 × 10^5^ PFU of MHV-68. Representative images (**a**) and quantitative analysis (**b**) of NLRP3 (red) and ASC (green) in lung tissues were measured by immunofluorescence staining at 3 days postinfection. DAPI was used to nuclear staining (blue). Scale bars, 200 μm. Protein representative bands (**c**) and quantitative analysis (**d**) related to NLRP3 activation were detection and analysis. Data are expressed as mean ± SEM, *n* = 6. *P < 0.05; **P < 0.01; ***P < 0.001; ****P < 0.0001; N.S., not significant.

**Fig. 8:**
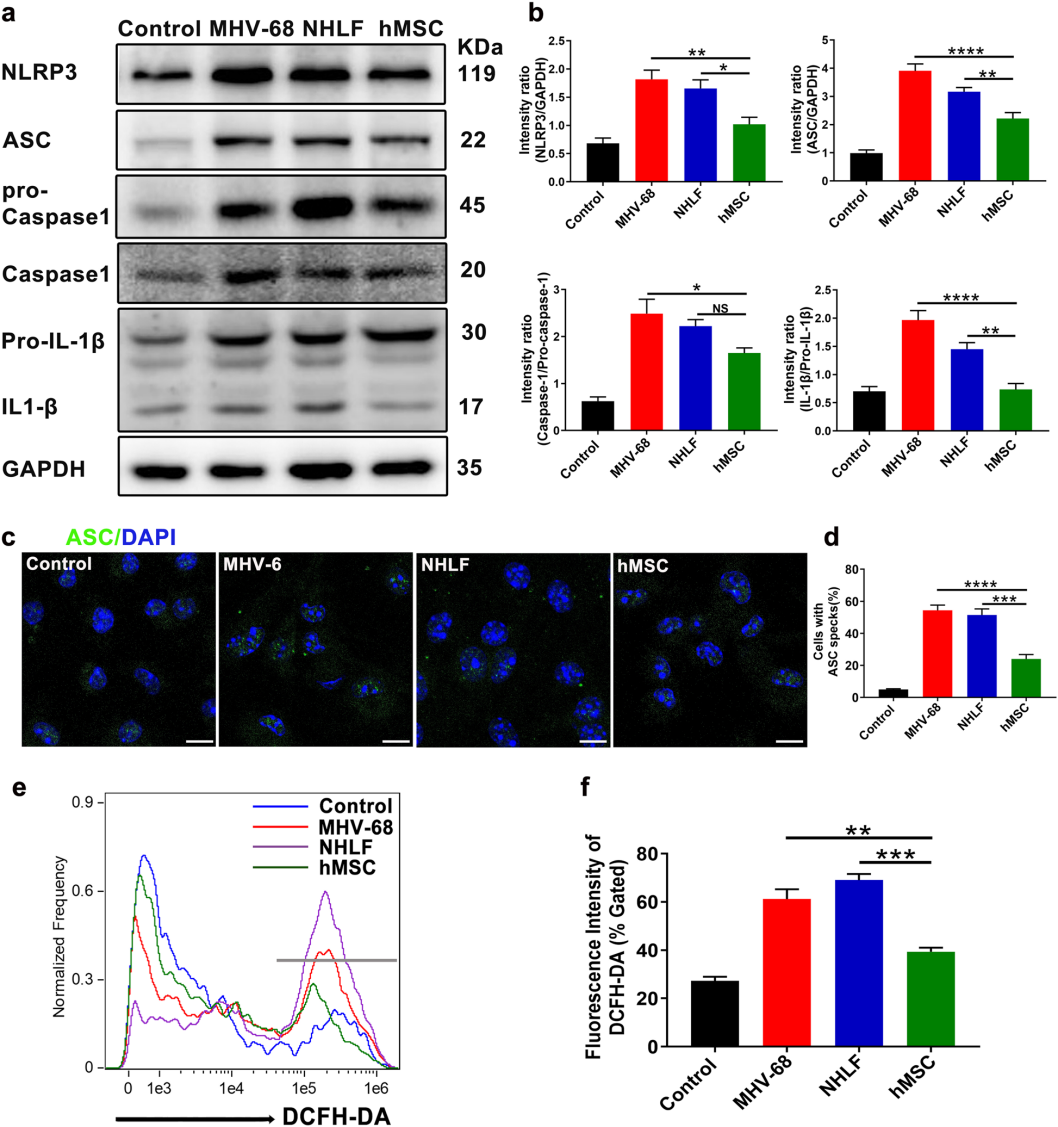
hMSCs reduces the NLRP3 activation in MHV-68-infected BMDMs. BMDMs (1 × 10^6^) were co-cultured with hMSCs (2 × 10^5^) for 48 hours after MHV-68 infection (MOI = 0.05). Protein representative bands (**a**) and quantitative analysis (**b**) related to NLRP3 activation were detection and analysis. **c** Representative images of ASC specks were detected by immunofluorescence assay in BMDMs infected by MHV-68. Scale bar: 5μm. **d** Percentage of cells containing an ASC speck in c. **e** The production of intracellular ROS was measured by flow cytometry. **f** Relative fluorescence intensity of DCFH-DA was analyzed. Data are expressed as mean ± SEM, *n* = 4. *P < 0.05; **P < 0.01; ***P < 0.001; ****P < 0.0001; N.S., not significant

Previous studies showed that MHV-68 induced IL-1β secretion in a manner dependent on NLRP3 and ASC [32, 33]. To determine whether hMSCs infusion affects NLRP3 activation, we examined the expression of corresponding components related to NLRP3 signalling pathway. Firstly, we detected the expression of NLRP3 and ASC in lung tissues on day 3 post-infection by immunofluorescence staining (Fig. 7a and b). Results showed that hMSCs infusion significantly reduced NLRP3 activation, including decreased expression of NLRP3 and ASC protein. Western blot results from the lung homogenate also showed similar results, including decreased levels of NLRP3 and ASC, mature IL-1β and cleaved caspase-1 (p20) in hMSCs-infusion group, instead of NHLFs’ (Fig. 7c and d). Using the same transwell system, we found reduced NLRP3 activation in BMDMs after co-culture with hMSCs (MOI = 0.05), consistent with the results in vivo (Fig. 8a and b). Moreover, we detected ASC speck formation which is an important step of NLRP3 inflammasome activation [34]. Confocal fluorescence microscopy showed the fewer numerous ASC speckles in co-culture group with hMSCs other than NHLFs’ group (Fig. 8c and d).

Production of ROS is especially important as a central and common upstream of cellular signals for NLRP3 inflammasome activation. So, we used the cell permeablesubstrate DCFH-DA to detect the ROS generation in BMDMs by flow cytometry. As presented in Fig. 8e and f, the cellular ROS fluorescence intensity was significantly decreased in hMSCs co-culture group, compared to the NHLFs’ group. These results suggested that hMSCs attenuated NLRP3 activation maybe by suppressing ROS production in macrophages.

**Fig. 9:**
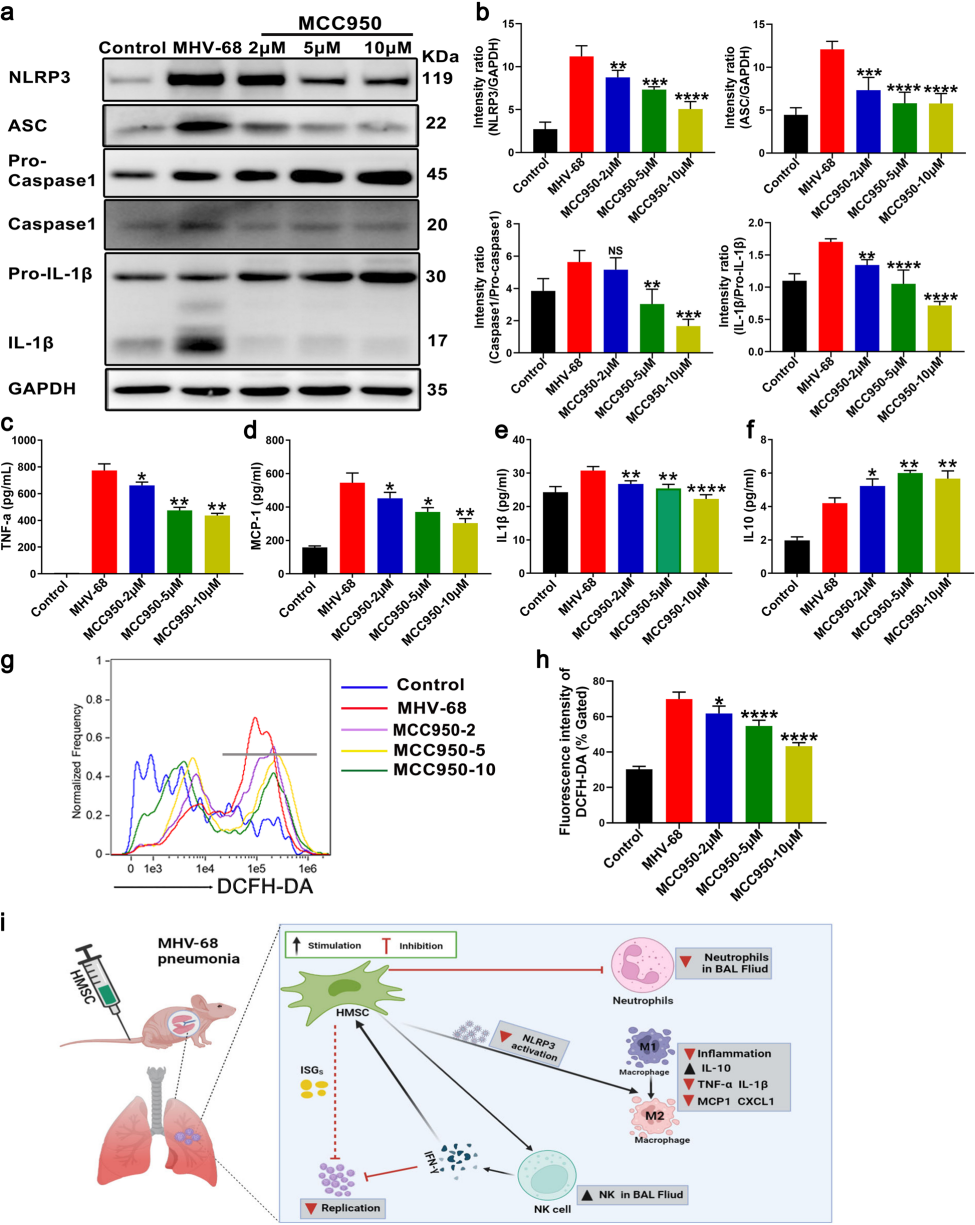
The effects of NLRP3-inflammasome inhibitor MCC950 on MHV68-infected BMDMs. BMDMs (1 × 10^6^) were treated with MCC950 (2μM, 5μM, and 10μM) for 24 hours after MHV-68 infection (MOI = 0.05). Protein representative bands (**a**) and quantitative analysis (**b**) related to NLRP3 activation were detection and analysis. The level of cytokines, i.e. TNF-α (**c**), MCP-1 (**d**), IL-1β (**e**) and IL-10 (**f**) in supernatant from BMDMs were measured and analyzed by ELISA. **g** ROS production in BMDMs was measured by DCFH-DA staining. **h** Relative fluorescence intensity of DCFH-DA was analyzed. **i** Schematic illustration of how hMSCs lessen the severity of MHV-68 pneumonia. Data are expressed as mean ± SEM, *n* = 4. *P < 0.05; **P < 0.01; ***P < 0.001; ****P < 0.0001; N.S., not significant

In addition, we used a selective small-molecule NLRP3-inflammasome inhibitor MCC950, to verify NLRP3 activation in the role of MHV68 pneumonia. Treating BMDMs with MCC950 dose-dependently inhibited the level of NLRP3 and ASC after MHV68 infection (MOI= 0.05), as well as caspase-1 and IL-1β activation (Fig. 9a and b). The level of pro-inflammatory cytokines, i.e. TNF-α, IL-1β and MCP-1 was decreased and anti-inflammatory cytokine IL-10 was increased in BMDMs (Fig. 9c-f). Moreover, ROS production in BMDMs was significantly reduced after MCC950 treatment (Fig. 9g and h). These data strongly supported therapeutic effects of hMSCs on MHV-68-induced pneumonia were partly due to downregulated NLRP3 activation in vivo and in vitro (Fig. 9i).

## Discussion

Epstein-Barr virus (EBV) is well-known for being a carcinogen. Besides that, pneumonia is also a common symptom of EBV infection, but lack of sufficient studies [35, 36]. In our study, we used the EBV-related MHV-68 mouse model to investigate the histological changes associated with interstitial pneumonia. Infected BALB/c-nu mice exhibited obvious simplification of alveoli, thickened alveolar septa, interstitial oedema and inflammatory cell infiltration. This well-established mouse model of MHV-68 pneumonia provides a valuable tool for drug screening and strategy development.

Using this model, we demonstrated that systemically intravenous human bone marrow-derived MSCs (hMSCs) partially protect the lung architecture against MHV-68-induced injury. MSCs have been revealed for their anti-pneumonia potential in various models, such as lung injuries induced by bleomycin, monocrotaline, amiodarone, *E. coli* bacteria or lipopolysaccharide (LPS) [8–11, 16]. Our data add great value to support MSCs treatment as an efficacious therapy for MHV-68 pneumonia.

A single bolus injection of hMSCs has been found to provide significant protection against pneumonia, reducing alveolar simplification and inflammatory cell infiltration. Understanding the mechanism behind this protective effect is crucial for the future application of MSCs treatment.

Firstly, the protective effect of MSC is primarily attributed to their ability to target macrophages and monocytes, which play a key role in the production of inflammatory mediators during inflammatory diseases. Previous studies on other pneumonia models have reported that MSCs moderate the inflammatory response by shifting macrophages from a proinflammatory (M1) to an anti-inflammatory (M2) phenotype through the secretion of various factors or modulating signal pathways [16, 37]. François *et al.* also reported that co-culture of human MSC and CD14^+^ monocytes induced an M2-like macrophage phenotype, characterized by anti-inflammatory properties and more potent phagocytic activity [38]. In a mouse model of ischemia/reperfusion (IR)-induced liver sterile inflammatory injury, Li and colleagues demonstrated that MSCs infusion protected hepatocellular from damage, shifted macrophage polarization from M1 to M2 phenotype, and reduced inflammatory mediators [18]. In the ARDS environment, MSCs and their extracellular vesicles (EVs) induced changes in macrophage phenotype by enhancing macrophage oxidative phosphorylation [39]. The above mentioned MSCs actions are consistent with our findings of hMSCs that promoted M2 transformation in MHV-68-infected lung and BMDMs. Moreover, MSC’ action on M1/M2 polarization is probably through a paracrine mechanism, as evidenced by our transwell experiments that blocked direct contact. In addition, many studies showed that M2 macrophages promoted the development of tissue fibrosis through secreting IL-10 and TGF-β [30, 40], whereas our results showed an anti-fibrosis effect after hMSCs infusion. We considered the possibility that hMSC treatment may reduce excessive immune responses to MHV-68 infection. Therefore, the MSC properties highlight the importance of maintaining balance in macrophage M1/M2 polarization, as any imbalance may have detrimental effects resulting in varied diseases or states of inflammation.

Secondly, hMSCs administration could sufficiently inhibit the activation of inflammasomes pneumonocytes. Inflammation is a protective immune response that is mounted by the innate immune system in response to harmful stimuli such as pathogens, dead cells, or irritants. However, an exaggerated immune response and inflammation during infections cause tissue damage, which is a major contributor to infectious disease-induced mortality. Numerous studies have demonstrated significant inflammasome activity in viral infection models, including the activation of caspase-1 and the secretion of interleukin-1β (IL-1β) [32, 33, 41]. Of particular note, A recent study has shown that caspase-6, an important component of the inflammasome, promotes the activation of programmed cell death pathways such as pyroptosis, apoptosis, and necroptosis (PANoptosis), and plays a critical role in host defense against influenza A virus (IAV) infection [42]. In our study, the inflammasome from macrophages and lung homogenates observed a significant activation, accompanied by increases in ROS production and IL-1β secretion. Our MSC treatment effectively inhibited the inflammasome, thus alleviating the symptoms of pneumonia, which further proved that lung damages in MHV-68 pneumonia are due to the activation of inflammasomes in pneumonocytes. The NLRP3-inflammasome inhibitor MCC950 further confirmed this effect. Our results suggest that NLRP3 inflammasomes play pivotal roles in the pathogenesis of pneumonia, and modulation of its signalling pathways may provide a possible targeting strategy in MSC-mediated immune regulation against MHV-68-induced pneumonia, as well as other inflammasome activation related pneumonia. It is worth noting that MSC does not always inhibit inflammation. In some cases, MSCs could promote inflammation when there are only low levels of inflammation signals (such as TNF-α and IFN-γ), and the immune system is underactivated, which can restrain inflammation [9, 21, 43].

Thirdly, MSCs possess broad immunoregulatory properties that extend to both innate and adaptive immune systems. In cases where the immune system is overactivated, MSCs can switch overactivated immune cells from a pro-inflammatory phenotype to an anti-inflammatory phenotype. This results in the suppression of immune effector cells and the activation of immune suppressor cell. Innate immune responses against EBV can be triggered not only in its main target cells, but also in other cells of the innate immune system [22–25]. As shown in our data, MHV-68 infection activated the innate immune responses marked by the infiltration of immune cells at the site of infection, including macrophages, neutrophils and NK cells in MHV-68-infected lung (Fig. 4). Treatment with hMSCs significantly altered the number and secretory function of these immune cells, as reflected in the secretion of cytokines and chemokines, such as IFN-γ, TNF-α, IL-6, IL-1β, IL-10, MCP-1 and CXCL1 (Fig. 2).

Fourthly, in terms of the potential direct effect of MSCs against viruses, we observed a reduced pulmonary viral load following MSC treatment. Compared to more differentiated cells, MSCs are typically more resistant to viral infections due to the presence of IFN-stimulated genes (ISGs) that can target various stages of the viral cycle. This mechanism prevents viruses from overpassing the cell membrane, blocking the endocytic route, nuclear import and transcription of mRNAs, translation of protein, genome integration/amplification, virus assembly, and release [44–46]. Although we can’t exclude the possibility that MSC treatment dilutes the local suitable host cell intensity for the infection, we believe IFN may play a significant role in this effect. In most cells, interferon (IFN) response is a major first line of defense against viral infection. The antiviral activity of IFN-γ against several herpesviruses has been demonstrated for human EBV (HEBV), MHV-68, human cytomegalovirus, varicella-zoster virus, herpes simplex virus-1 (HSV-1), and HSV-2 [44–50]. Our previous study also found that IFN-γ-stimulated guanylate-binding protein 1 (hGBP1) from human MSCs could protect against *Toxoplasma gondii* infection [28]. One evidence supporting this thought is the uplifted IFN-γ levels in MHV68-infected lungs in our study; we speculate there may be intrinsically expressed ISGs in hMSCs involved in the antiviral protective action. By examining published IFN-γ stimulated RNA-seq data on “defense response to virus” gene ontology terms (GO database: 0051607), we identified numerous upregulated ISGs in hMSCs, including APOBEC3G、BST2、IFIT1-3、IFITM-1、SAMHD1, and TRIM22. (Supplementary Fig. 4). Therefore, it is highly likely that ISGs in hMSCs are involved in the antiviral effect of hMSC. However, it is unclear whether one or more of these ISGs work together against MHV-68 pneumonia. Future studies are needed to determine whether these ISGs contribute directly to MHV-68 replication or if they act by inhibiting some stage of the virus cycle.

Research into stem cell and progenitor cell–based therapies has yielded promising results for immune-mediated and inflammatory diseases [8–21]. In our system, the beneficial response appears to be stem cell–specific, as we did not observe any effect with human lung fibroblasts (NHLFs). Mesenchymal stromal cells (MSCs) are multipotent progenitor cells that can be derived from various adult tissues. They possess immune modulation functions on multiple immunocytes, including innate and adaptive immunity. MSCs are responsive to the host microenvironment and are capable of adopting a proinflammatory or anti-inflammatory phenotype by interfering with innate and adaptive immune responses both in vitro and in vivo [9, 21, 43]. MSCs have been widely used for multiple clinical applications, including autoimmune and inflammatory diseases, ARDS, allotransplant rejection, spinal cord injuries, myocardial infarction, degenerative disorders, bone diseases and more. Additionally, MSCs have antiviral properties and have been utilized in treating various viral infections in recent years. Notably, there has been a rapid increase in the number of MSC-based therapies for COVID-19, as MSC transplantation has improved the outcomes of seven enrolled patients with COVID-19 pneumonia in Beijing YouAn Hospital, China [51]. Despite limited published clinical data, the clinical trials conducted thus far have reported that MSC application in viral diseases is safe [9, 19, 52, 53].

## Conclusions

In the present study, we have observed that the administration of hMSC could effectively enhance tissue regeneration, mitigate inflammation, and significantly reduce the viral load in the lungs (as depicted in Fig. 9i). These findings hold great promise for the development of a novel therapeutic strategy for the treatment of lung injury induced by MHV-68. Moreover, the potential application of MSC-based cell therapy in preventing the onset of pneumonia associated with Epstein-Barr virus (EBV) warrants further investigation.

## Materials and methods

### Mice

Pathogen-free C57BL/6J mice weighing between18–22 g and female BALB/c nude mice weighing between 13–16 g were purchased and housed in the pathogen-free mouse room at the experimental animal center of Guangzhou Medical University. The animal use protocol has been reviewed and conducted under the supervision of the Experimental Animal Ethics Committee of Guangzhou Medical University in Guangzhou, China.

### Cells culture

The murine fibroblast cell line NIH/3T3 cells and normal human lung fibroblasts (NHLF) were procured from the American Type Culture Collection (Manassas, VA). Both NIH/3T3 and NHLF were cultured in Dulbecco’s modified Eagle’s medium (DMEM) (Gibco, Invitrogen, Carlsbad, CA, USA) supplemented with 10% fetal bovine serum (FBS) (Gibco), 2 mM glutamine, and 100 U of penicillin/streptomycin per ml.

Human bone marrow-derived MSC (hMSC) were obtained and cultured as previously described [28]. hMSCs were isolated from bone marrow aspirates (10-30 ml) of healthy voluntary donors after obtaining informed consent and in accordance with guidelines approved by the Ethics Committee of Sun Yat-Sen University (2018-132). The expression of surface markers in our MSC preparations was tested via flow cytometry (BD LSRII; BD Biosciences) using α-CD29 (BD #559883), α-CD44 (BD #560531), α-CD73 (BD #550257), α-CD90 (BD #561558), α-CD105 (BD #560819), α-CD166 (BD #559263), α-CD34 (BD #555821), α-CD31 (BD #555446), and α-CD45 (BD #555482) (BD Biosciences, Franklin Lakes, NJ, USA), as well as their differentiation abilities into osteoblasts and adipocytes. All MSCs used in this study were at passage 3 to 8. Cellular controls were normal human lung fibroblasts (NHLFs).

Bone marrow-derived macrophages (BMDMs) were collected using the previously described method [29]. Briefly, femurs and tibia were flushed with cold sterile phosphate-buffered saline (PBS) to isolate bone marrow cells, . Which were then subjected to red blood cell lysis. After washing twice with PBS, the cells were resuspended in DMEM:F-12 supplemented with 10% FBS, 20ng/mL of murine M-CSF (#315-02-50, PeproTech), and 100 U of penicillin/streptomycin per ml, The cells were then plated and cultured in 12- or 6-well plates for 6-7 days until further experiments. For co-culture experiments, hMSCs were seeded at a density of 10,000/cm^2^ in 6-well plates or transwells, and cultured in hMSC growth medium for 48 h. Prior to co-culture and MHV-68 infection, hMSCs were washed three times with PBS and co-cultured with BMDMs in the macrophage culture medium.

### Preparation of virus and infections

A recombinant murine gammaherpesvirus 68 (MHV-68) expressing the firefly luciferase gene under the control of the viral M3 promoter (M3FL) was constructed by Hwang et al. [7]. MHV-68 was grown and purified on NIH/3T3 cells. Briefly, NIH/3T3 cells were seeded in complete DMEM and infected with MHV-68 at a multiplicity of infection (MOI) of 0.1 for 3-4 days. The culture supernatants were then filtered through a 0.45 um filter and virus particles were pelleted by ultracentrifugation through a 30% sucrose cushion (25 000 g for 3 h at 4°C, Beckman SW32Ti rotor). Aliquots of virus were stored at -80˚C and viral titers were measured by plaque assay of serial dilutions in NIH/3T3 cells after fixation with 2% crystal violet in 20% ethanol.

For in vivo infection, frozen MHV-68 was thawed on ice and female Balb/c-nu mice were intranasally inoculated with either MHV-68 (2 × 10^5^ PFU) in 40 μl of PBS or solvent PBS after being anesthetized by intraperitoneal injection of a mixture of Ketamine and Xylazine (100 mg/Kg and 2 mg/Kg). Following infection, the body weight and survival of mice were monitored and recorded every other day.

For in vitro infection, BMDMs alone or BMDMs accompanied with hMSCs were infected with MHV-68 at MOI = 0.05 for 48 h. Supernatants were collected to measure viral titer as above and the expression of cytokines by ELISA. Samples were harvested for RT-PCR, western blot, and flow cytometry analysis.

### Bronchoalveolar lavage

At the designated time, the mice were euthanized according to the aforementioned protocol, and the trachea was exposed. A 20-gauge angiocatheter was inserted into the trachea, and the lungs were flushed with three separate 0.8 ml volumes of sterile PBS. The bronchoalveolar lavage (BAL) fluid was collected, pooled, and centrifuged at 500 × g for 5 minutes at 4°C to pellet the cell fraction. The BAL fluid was stored at -80°C for PFU and ELISA assay, while the cell pellet was resuspended in cold PBS for flow cytometry analysis, utilizing Beckman Coulter equipment from the United States.

### Noninvasive imaging

In vivo bioluminescence imaging was performed using an optical IVIS system (Xenogen Corp., Alameda, CA), following the previously described protocol [7]. Female BALB/c mice were intranasally infected with 2 ×10^5^ PFU of M3-FL/MHV-68 or solvent PBS. At the designated time post-infection, the mice were intraperitoneally administered with 10 μl/g weight of D-luciferin (Storage concentration: 15mg/mL. Xenogen Corp.), The D-luciferin was converted into oxyluciferin, which emitted photons detected by a charge-coupled device (CCD) camera at 562 nm in the IVIS Spectrum system (Xenogen, Alameda, CA, USA).

### Enzyme-linked immunosorbent assay (ELISA)

The excised lungs were weighed, washed and homogenized in PBS. The expression of cytokines and chemokines,including TNF-α, IFN-γ, IL-10, IL-1β, MCP-1, IL-6, IFN-α (Neobioscience, Beijing, China), and CXCL1 (R&D Systems),was assayed in the supernatants from lung homogenates and BAL fluid using ELISA. The manufacturer's recommendations were followed during the assay.

### FACS analysis

Surface markers of BMSC and BAL fluid were analyzed using flow cytometry. BAL cells were obtained by centrifugation of pooled BAL fluid, and the resulting cell pellet was stained with fluorochrome-labelled antibodies to obtain a leukocyte differential, including neutrophils (Ly6G+CD11b+; α- Ly6G, Clone: RB6-8C5 25-5931-82, eBioscience; α-CD11b, Clone: M1/70, BD #557396), macrophages (Ly6G−CD11b+F4/80+; α-F4/80, Clone: APC- 20-4801-U100, eBioscience), and natural killer cells (CD3−CD49b+; α-CD3e, Clone: 145-2C11, #17-0031-81, eBioscience; α-CD49b, Clone: DX5, #563063, BD). Total cell numbers were obtained using an automated cell counter (JIMBIO, China) prior to flow cytometry, following the manufacturer’s recommendations. In some experiments, the expression of CD80 (α- CD80, Clone: 16-10A1, #11-0801-81, eBioscience) and CD206 (α- CD206, Clone: C068C2, #141716, Biolegend) from BAL and bone marrow-derived macrophages (BMDMs) were detected. CytoFLEX (Beckman Biosciences) and FlowJo 7.6 software (Tree Star, Ashland, OR, USA) were used for analyses.

Intracellular staining of IFN-γ (α- IFN-γ, Clone: XMG1.2, #12-7311-82 ), in BAL cells was performed by stained for cell surface markers, followed by fixation and permeabilization using a fixation/permeabilization kit (eBioscience). Flow cytometry data were analyzed using the aforementioned method .

To detect intracellular ROS, BMDMs with stained with 2',7'-Dichlorodihydrofluorescein diacetate (DCFH-DA, Beyotime, China). BMDMs were co-cultured with hMSCs or treated with varying concentrations of MCC950 in the macrophage culture medium for 48 h or 24 h after MHV-68 infection (MOI = 0.05). Subsequently, DCFH-DA (10 μM) were added to the cells for 20 min in dark at 37°C, Then cells were then harvested, and the level of intracellular ROS was using flow cytometry (AmnisImage StreamXMarkII, Merk).

### Histological analysis and immunofluorescence staining

The lungs were removed from the animals in an unmanipulated and noninflated state and fixed in 4% buffered paraformaldehyde solution, The fixed lungs were then embedding in paraffin, and 4-µm mid-modiolar sections were cut and processed for Hematoxylin/Eosin (H&E) or Masson and/or immunofluorescence staining. Hematoxylin/Eosin (H&E) and Masson staining were performed by serviceBio., WuHan, China. The H/E-stained slides were evaluated for viral-induced lung pathology with a Leica (DMi8) microscope. The percentage of airspace and acinar tissue were calculated in each slide from each group of mice using Image J software. For each mouse, 10 randomly selected high-power fields were assessed. and the investigators were blinded to group allocation.

For immunofluorescence staining, sections were placed on glass slides (Bio-Optica), blocked for 30 minutes with PBS-Tween 0.05% plus 0.5% FBS, and then incubated for 18 hours at 4°C with combinations of primary antibodies to α-SMA (1:500, #19245T), NLRP3 (1:500, # DF7438) and ASC (1:500, # sc-514414), After incubation, sections were washed three times in PBS, incubated at room temperature for 1 hour with combinations of Alexa Fluor 488- or Alexa Fluor 647-conjugated donkey anti-rat IgG pAb (Servicebio), and then washed, counterstained with DAPI (4-,6-diamidino-2-phenylindole), and mounted in Prolong Gold antifade reagent (Life Technologies). Fluorescence was visualized with a Zeiss LCM880 confocal microscope (Oberkochen, Germany) and analyzed with Zen imaging software or ImageJ software.

### Real-time reverse transcription-PCR (RT-PCR)

Total RNA was extracted from lung tissues or BMDMs at different time points using Trizol reagent (Invitrogen, Carlsbad, CA) following the manufacturers’ instructions. The expression levels of the viral immediate-early ORF50 gene were measured using QuantiTect SYBR Green PCR Master Mix (Takara, Kyoto, Japan) and RT-PCR analysis. The primers used for ORF50 were as follows: forward primer GATTCCCCTTCAGCCGATAAG, and reverse primer CAGACATTGTAGAAGTTCAGGTC. Real-time PCR reactions were carried out using a LightCycler480 instrument (Roche, Indianapolis, IN, USA). The relative mRNA levels were calculated by normalization to glyceraldehyde-3-phosphate dehydrogenase (GAPDH) using the following primers: forward primer 5’-CCGCGTTCTTCCATTTGTGT-3’, and reverse primer 5’-ACATGATTTCGCATTTCGTCAT-3’).

### Western blot analysis

Immunoblotting was conducted according to previously described methods [28, 54]. Briefly, lung tissues or BMDMs samples were lysed with RIPA buffer containing a protease inhibitor mixture. The supernatants were collected and quantified using a BCA protein quantitative Kit from ThermoFisher Scientific, USA. The lysates were boiled for 10 minutes, and equal amounts of protein were separated by SDS-PAGE and transferred to a polyvinylidene fluoride (PVDF) membrane. The membrane was blocked and incubated with primary antibody overnight at 4˚C, followed by a secondary antibody conjugated with horseradish peroxidase at room temperature for 1 hour. Glyceraldehyde 3-phosphate dehydrogenase (GAPDH) was used as loading control. The levels of proteins were assayed using primary antibodies, including α-ARG1 (1:1,000, #DF6657), α-NOS2 (1:1,000, #AF0199), α-NLRP3 (1:1,000), α-ASC (1:1,000) , α-Caspase 1 (1:1,000, AF5418), α-IL-1β (1:1,000, #AF5103), and α-GAPDH (1:2,000, #TA-08, ZSGB-BIO, BeiJing China). Antigen-antibody complexes were detected by enhanced chemiluminescence (GE Amersham Imager 600, USA). Band intensities were quantified using ImageJ software from (NIH) and standardized with respect to GAPDH levels.

### Statistical analysis

The data obtained from the study were analyzed using GraphPad Prism 7 software (GraphPad, San Diego, California). In the case of in vivo experiments, the unpaired two-tailed Student’s t-test was used to test for statistical significance between groups. For in vitro experiments, data were collected from at least three independent experiments. The quantitative data are presented as means ± SEM, and statistical differences was considered at *p < 0.05, **p < 0.01, and ***p < 0.001. The abbreviation NS was used to indicate no significant difference.

### Supplementary Information

**Additional file 1:** **Supplementary Fig 1.** Establishment of MHV-68 infected-nude mice pneumonia model. **Supplementary Fig 2.** The phenotype and differentiation capacity of hMSCs. **Supplementary Fig 3.** hMSCs regulated macrophage polarization in MHV-68-infected BMDMs. **Supplementary Fig 4.** RNA-seq data analysis. Human MSCs from three different donors were pretreated for 12 h with IFN-γ (20 ng/mL).

## Data Availability

All data generated or analyzed during this study are included in this article.
