# Supplementary material for: hMSCs treatment attenuates murine herpesvirus-68 (MHV-68) pneumonia through altering innate immune response via ROS/NLRP3 signaling pathway
Source: Mol Biomed. 2023 Sep 14;4:27. doi: 10.1186/s43556-023-00137-z (PMC10499773; doi:10.1186/s43556-023-00137-z)

Supplementary Fig 1.

Establishment of MHV-68 infected-nude mice pneumonia model.

Mice were infected intranasally with 2 × 10^5^ PFU of M3FL. (a) In vivo bioluminescent imaging showing the in situ localization of luciferase signal after intranasal infection at the indicated day. (b and d) The expression of viral immediate-early ORF50 gene and the titer of infectious virus were determined by RT-PCR (b) and plaque assay (d), respectively. (c) H&E-stained sections of MHV-68-infected lung at the indicated day post-infection. Scale bars, 200 μm.


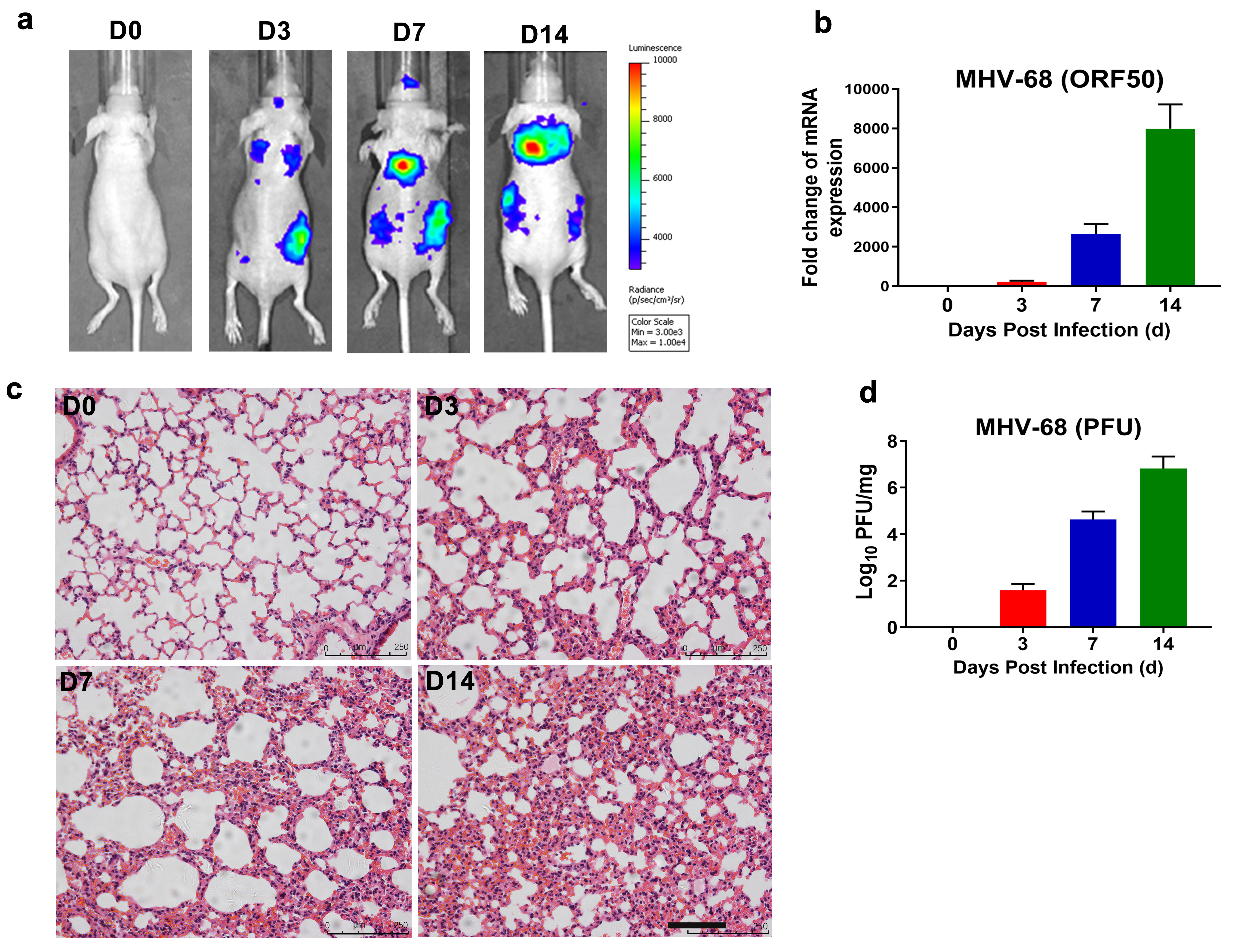


Supplementary Fig 2.

The phenotype and differentiation capacity of hMSCs.

(a) Representative flow-cytometric analysis of hMSCs. CD34, CD45, CD44, CD29, CD105, CD73, CD31, CD90, and CD166 expression in hMSCs were analyzed by flow cytometry. (b) Oil red O staining of adipogenic-differentiated hMSCs. (c) Alizarin Red S staining of osteogenic-differentiated hMSCs. Scale bars, 200 μm.


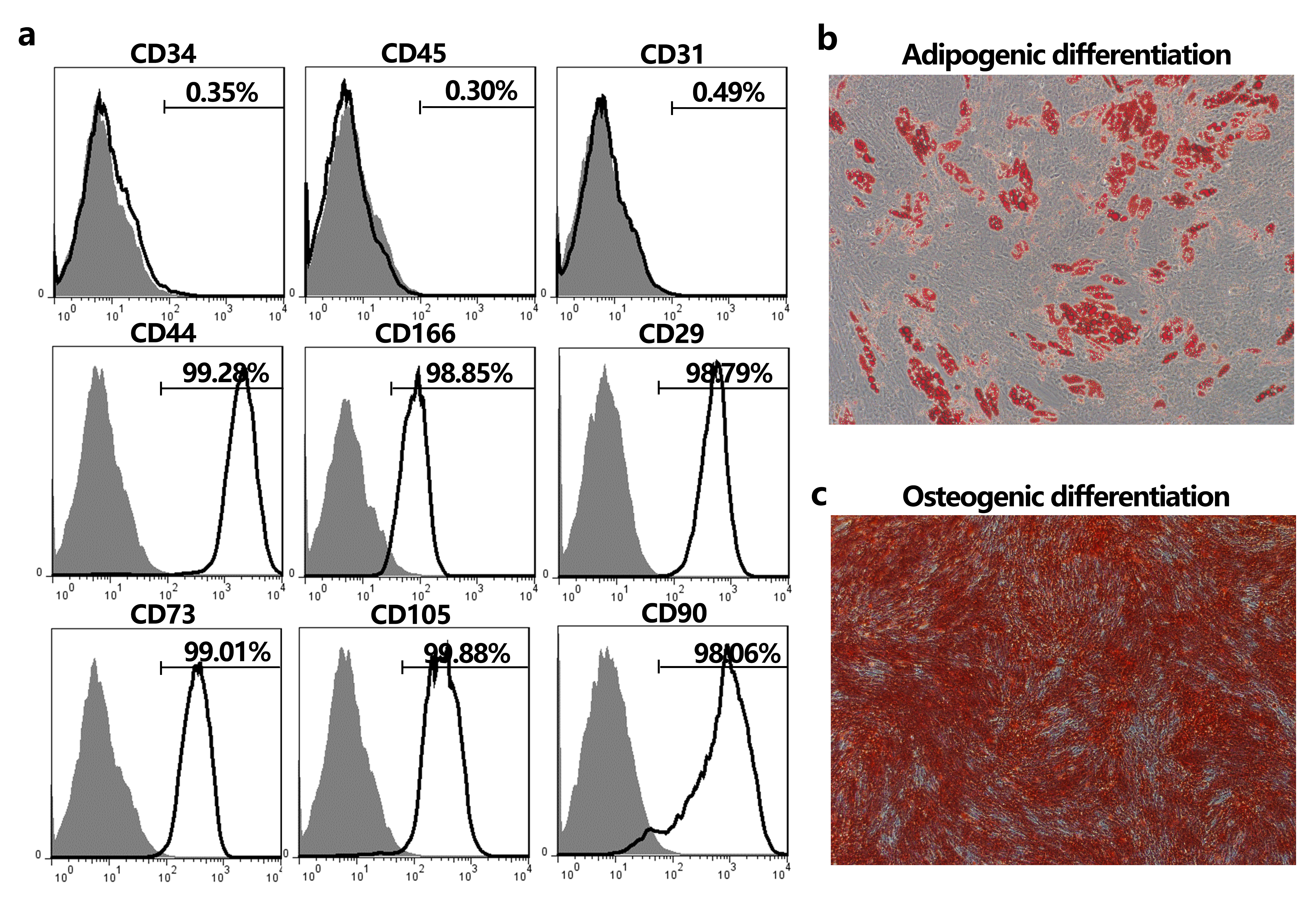


Supplementary Fig 3.

hMSCs regulated macrophage polarization in MHV-68-infected BMDMs.

BMDMs (1 × 10^6^) were co-cultured with hMSCs (2 × 10^5^) for 48 hours after MHV-68 infection (MOI = 0.05). (a & b) The expression of ARG1 and NOS2 from co-cultured BMDMs were measured by Western blot. (c & d) The expression of CD80 (c) and CD206 (d) gated on CD11b^+^F4/80^+^ cells from co-cultured BMDMs were measured and assayed by flow cytometry (Left, representative flow cytometry data; Right, statistical analysis results). Data are expressed as mean ± SEM, n = 4. *P < 0.05; **P < 0.01; ***P < 0.001; ****P < 0.0001; N.S., not significant.


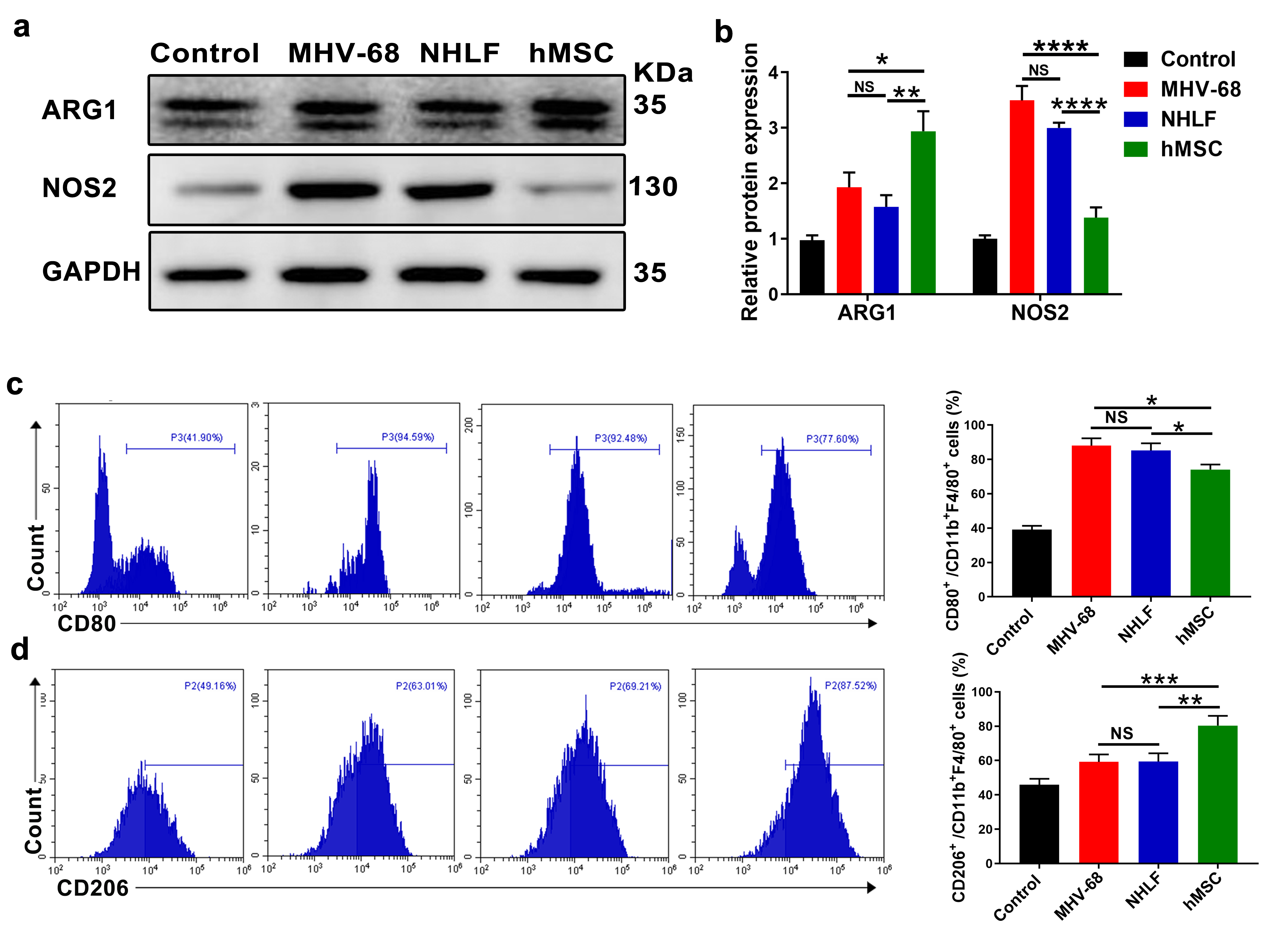


Supplementary Fig 4.

RNA-seq data analysis. Human MSCs from three different donors were pretreated for 12 h with IFN-γ (20 ng/mL). Then, RNA library preparation and sequencing were performed as recommended by the manufacturer. Sequencing data were processed using Consensus Assessment of Sequence and Variation using the default settings (CASAVA, version 1.8.2; Illumina). Genes with an RPKM value more than five were enrolled in the functional analysis according to the Gene Ontology (GO database: 0051607) database. Heat map displays of genes related to host defense to virus are shown. The heatmaps of these genes were drawn with the RPKM value taking the logarithm of two.


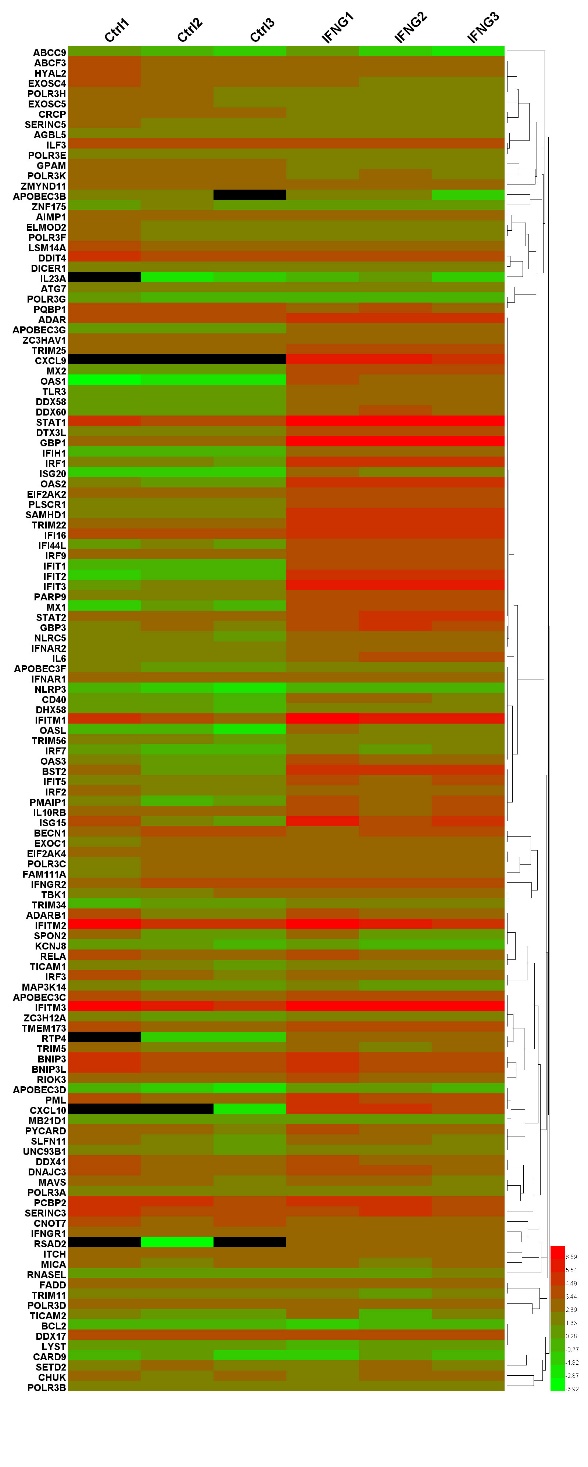

Supplement: Supplementary file 1 — Additional file 1: Supplementary Fig 1. Establishment of MHV-68 infected-nude mice pneumonia model. Supplementary Fig 2. The phenotype and differentiation capacity of hMSCs. Supplementary Fig 3. hMSCs regulated macrophage polarization in MHV-68-infected BMDMs. Supplementary Fig 4. RNA-seq data analysis. Human MSCs from three different donors were pretreated for 12 h with IFN-γ (20 ng/mL). [file 43556_2023_137_MOESM1_ESM.docx]
